# Medium-term storage of calf beddings affects bacterial community and effectiveness to inactivate zoonotic bacteria

**DOI:** 10.1371/journal.pone.0295843

**Published:** 2023-12-15

**Authors:** Delphine Rapp, Colleen Ross, Vanessa Cave, Paul Maclean, Ruy Jauregui, Gale Brightwell

**Affiliations:** 1 Food System Integrity, AgResearch Ltd, Hopkirk Research Institute, Palmerston North, New Zealand; 2 Data Science Team, AgResearch Ltd, Ruakura Research Centre, Hamilton, New Zealand; 3 Data Science Team, AgResearch Ltd, Grasslands Research Centre, Palmerston North, New Zealand; 4 New Zealand Food Safety Science & Research Centre, Hopkirk Research Institute, Palmerston North, New Zealand; Beni-Suef University: Beni Suef University, EGYPT

## Abstract

Land-spreading of animal faecal wastes -such as animal beddings- can introduce zoonotic enteropathogens into the food system environment. The study evaluated the effectiveness of animal beddings naturally contaminated by calf manure to reduce *E*. *coli* O157:H7 or *Salmonella enterica*. The two pathogens were introduced separately as a four strains-cocktail and at high (>6.5 Log_10_ g^-1^) concentration into bedding materials, and their inactivation over a 10 weeks-period was monitored by using a Most Probable Number (MPN) enumeration method. Inactivation of *E*. *coli O157*:*H7* was more effective in the bedding inoculated immediately after collection from calf pens than in the beddings inoculated after a 2 months-pre-storage period: *E*. *coli* O157:H7 levels were reduced by 6.6 Log_10_ g^-1^ in unstored bedding (0.5 Log_10_ g^-1^ recovered; 95%CI: 0.0–1.2), and by 4.9 Log_10_ g^-1^ in pre-stored bedding (2.2 Log_10_ g^-1^ recovered; 95%CI: 1.5–2.8) with a significant (p<0.05) difference between unstored and pre-stored. *S*. *enterica* was inactivated less effectively as counts were reduced by one order of magnitude, with no significant difference in inactivation between unstored and pre-stored beddings. Low levels of naturally occurring *E*. *coli* O157 and *Salmonella* spp. were detected in the non-inoculated beddings, as well as in the straw prior to use in the animal facility. To better understand the possible biological processes involved, the bacterial community present in the beddings was characterised by short-read 16S rRNA sequencing. Pre-storage of the bedding affected the composition but not the diversity of the bacterial community. Analyses of the key bacterial phyla suggested that the presence of a diverse and stable bacterial community might facilitate inactivation of the introduced pathogens, and a possible role of bacterial orders associated with lignocellulolytic resources. Overall, the study contributed to the understanding of the fate of zoonotic bacteria introduced in animal beddings during storage and identified bedding storage practices pre-and post-use in animal facilities that could be important to prevent the risk of zoonosis dissemination to the environment or to the dairy herds.

## Introduction

World dairy and beef production has undergone significant growth in the last 20 years and is projected to keep growing globally over the next decade, due to high population growth in some areas and a desire for high-protein food [1]. This demand will likely be met by intensification of farm practices and increased reliance on housing systems with bedding materials on which the animals walk, stand, lie, defecate and urinate. In dairy housing systems, natural materials such as straw, woodchips, wood shavings or sawdust are commonly used as organic bedding materials [2, 3]. These materials ensure cow comfort and can also be recycled as agricultural soil amendments providing nutrients to plants and soils, reducing the need for synthetic fertilizers, and contributing positively to soil quality and crop health [3–5]. However, animal manure and used beddings soiled with animal feces and urine can also be a potential reservoir for enteric pathogens and, if not handled appropriately, pose a significant risk to animal and human health [6, 7]. Among the enteric pathogens commonly detected in used animal beddings are *Escherichia coli* O157:H7 and *Salmonella* spp. [8, 9]. These bacteria are leading causes of foodborne illness outbreaks associated with consumption of meat, milk or fresh produces contaminated with animal manure [10, 11]. They have also been identified in outbreaks associated with amendments of vegetable fields with used animal beddings, or with direct contact of humans with used animal beddings [12–14].

Alongside control of these pathogens in the animals themselves, pre-treatment of cattle waste -including used bedding materials- prior to recycling on-farm has been promoted to reduce the spread of enteric pathogens [15]. The liquid fraction of animal wastes can be separated from the solid fraction, which itself can be used immediately or further maturated through different methods, including stock-piling or composting. Stockpiling of soiled animal beddings, also known as “static piling” or “passive composting”, consists of heaping or stacking used bedding or separated solids, either inside or outside animal facilities [16]. It is a simple storage practice used routinely by many farmers [17]. Compositing, which requires actively incorporating dry matter, managing moisture content, and regular mixing/aerating in order to mature animal wastes into high-value soil amendments, has the disadvantage to be challenging technically and costly for farmers [17, 18]. Adequate pre-treatment of used beddings is critical for the farm sustainability, as unproper pre-treatment of used beddings before re-use or land spreading has been shown to increase the prevalence of zoonotic micro-organisms in dairy herds [19]. Maturation and recycling of used animal beddings raised questions around the effect of recontamination through addition of freshly soiled bedding to the storage heap. Stored manure undergoes degradation during their maturation, with accompanying variations in chemical and/or structural composition as well as in the predominant microorganisms [20, 21], and there is currently no information on the inactivation of zoonotic bacteria if re-introduced in the beddings during the storage and maturation period.

Inactivation of *E*. *coli* O157:H7 or *Salmonella* spp. during the storage of stockpiles or maturation of composts was reported to vary substantially among studies, ranging from weeks to months [22, 23]. The effects of the heaps physical and chemical characteristics and the atmospheric conditions on pathogen inactivation has received considerable attention, as reviewed by Ongeng et al [24]]. There are comparatively few reports and limited understanding of the biological mechanisms that account for inactivation of foodborne pathogens. For example, You et al. [25] indicated that indigenous but unidentified bacteria in manure can exert an antagonist effect on the persistence of pathogens. Franz et al. [26] showed that the overall survival time of *E*. *coli* O157:H7 in cattle manure was negatively correlated with the number of coliforms. Semenov et al. [27], who characterized the bacterial community in manure by DGGE analyses, could not establish a strong relationship between bacterial community composition and survival time of *E*. *coli* O157:H7.

The aims of this study were (1) to compare the inactivation of *E*. *coli* O157:H7 and *S*. *enterica* when introduced in animal beddings before or during storage; and (2) to better understand the possible biological processes involved in the inactivation of *E*. *coli* O157:H7 and *S*. *enterica* by characterizing the bacterial community present in the bedding. The bedding material was sourced from a calf rearing facility and consisted of straw naturally contaminated by dairy calves’ excreta (e.g., feces, urine, hair, saliva, etc.). Straw without excreta was used to investigate the effect of the bacterial community naturally present in the straw. We hypothesized that both the inactivation of the food-borne bacteria and the diversity and composition of the bacterial community would be different between the straw-based materials and with or without pre-storage.

## Materials and methods

### Bacterial strains

Eight strains (four *E*. *coli* O157:H7 and four *Salmonella enterica* subsp. Enterica) isolated from dairy animals (calf feces and dairy effluents) and their environment (calf bedding or bird droppings), and fully characterized by whole genome sequencing (BioProject PRJNA933223) were used for the study. Each strain was revived from cryobeads (Microbank^TM^, Ngaio Diagnostics, Nelson, New Zealand) using Sheep blood agar (Fort Richard Laboratories, Auckland, New Zealand) and Tryptic Soy Agar (TSA; Fort Richard Laboratories) at 35°C as previously described [28]. All the bacterial growth collected from TSA was resuspended into 50 ml of 0.85% saline. Bacterial cocktails for inoculation of bedding material were freshly prepared by combining each undiluted bacterial suspensions in equal proportions, resulting in a 120 mL of *S*. *enterica* cocktail and a 120 mL of *E*. *coli* O157:H7 cocktail. The concentration of each pure culture was confirmed by serial dilution in 0.1% peptone and enumeration by plate counts on Sheep Blood agar after 24-h incubation at 35°C.

### Bedding collection and processing

Two straw-based beddings were used for the experiment. The “straw + manure” bedding was sourced from one pen of the fully covered calf rearing facility of Dairy Farm 4 at Massey University (Palmerston North, New Zealand). Sampling did not require a field permit consent but complied with the farm’s health and safety requirements and biosecurity policies. The straw was collected by bulking bedding samples (200-400g each) from 40 locations in the pen, avoiding water and feed troughs, and from the top (5 cm) and bottom (25 cm) layers of the bedding. The pen from which bedding was collected housed 40 unweaned (<2-month-old) calves at collection time but had been used for the entire duration of the calving season (approximately 6 weeks; 100 calves in total). The “straw + manure” bedding contained straw, fresh calf urine and fecal material and had a low level of visible body material (such as calf hair). The “straw” was collected from the middle (one location) of an unopened bale stored in another covered shed at the same farm. Care was taken to avoid the outside layer, which was unprotected from the elements and from the birds. Both bedding materials were collected using sterile gloves. The collected bedding samples (~2 kg (wet weight) each) were immediately transported in insulated containers to the laboratory. The water content of the collected bedding sample estimated by gravimetry was 63% and 9% for the straw + manure and the straw, respectively. Each bedding sample was cut into 2–5 cm long pieces using sterile scissors and mixed thoroughly using the quarterly separation method described by Axmann et al. [29]. Each prepared bedding sample was then divided into two equal portions, one of which was used immediately (“unstored”) while the other was placed in a closed container stored in a controlled temperature (20°C) room for 2 months prior to microcosm preparation (“prestored”). Each bedding sample was regularly mixed manually during the storage period in order to limit heterogeneous development of anaerobic conditions.

### Bedding microcosms experimental design

Four types of bedding material (“unstored straw”, “pre-stored straw”, “unstored straw + manure” and “prestored straw + manure”) were used for preparing the bedding microcosms. The microcosms were prepared by placing 10 grams (wet weight) of bedding materials in a 720 ml whirl-pack® bag, followed by incorporation of either 1.0 ml of freshly prepared *S*. *enterica* cocktail or 1.0 ml of freshly prepared *E*. *coli* O157:H7 cocktail. In uninoculated (control) microcosms, 0.85% saline was added in place of the bacterial cocktail. In total, 154 microcosms were inoculated with *E*. *coli* O157:H7, 154 microcosms were inoculated with *S*. *enterica*, and 174 microcosms were uninoculated, giving a total of 482 independent microcosms. All inoculated and uninoculated microcosm bags were left slightly open for sufficient aeration and incubated in a dark and temperature-controlled (20°C) room. Microcosms were removed from the incubation room at the following intervals: 7, 13, 20, 35 or 70 days. At each sampling point, five to eight microcosms for each each inoculum and each bedding type were removed and immediately subjected to different analyzes (enumeration of *E*. *coli* O157:H7, enumeration of *Salmonella* spp., pH and moisture content) as listed in Table 1. In addition, at sampling points 0, 35 and 70 days, one to two microcosms for each inoculum and each bedding type were transferred into storage at -80°C until DNA extraction.

**Table 1:** Bedding microcosms experimental design with the number of microcosms used for each analyses.

Bedding type		Inoculum^a^	Sampling points (days of incubation)	No of microcosms analyzed at each sampling point				
material	pre-storage treatment			*E*. *coli* O157 counts	*Salmonella* counts	pH	moisture	Bacterial community
Straw	unstored	*E*. *coli* O157:H7	0, 13, 20, 35, 70	3	-	1	1	2
		*S*. *enterica*	0, 13, 20, 35, 70	-	3	1	1	2
		0.85% saline (control)	0, 13, 20, 35, 70	3 ^b^	3 ^c^	1	1	2 ^e^
	pre-stored	*E*. *coli* O157:H7	0, 7, 13, 20, 35, 70	3	-	1	1	2
		*S*. *enterica*	0, 7, 13, 20, 35, 70	-	3	1	1	2
		0.85% saline (control)	0, 7, 13, 20, 35, 70	3 ^c^	3 ^d^	1	1	2
Straw + manure	unstored	*E*. *coli* O157:H7	0, 13, 20, 35, 70	3	-	1	1	2
		*S*. *enterica*	0, 13, 20, 35, 70	-	3	1	1	2
		0.85% saline (control)	0, 13, 20, 35, 70	3 ^b^	3 ^c^	1	1	2
	pre-stored	*E*. *coli* O157:H7	0, 7, 13, 20, 35, 70	3	-	1	1	2
		*S*. *enterica*	0, 7, 13, 20, 35, 70	-	3	1	1	2
		0.85% saline (control)	0, 7, 13, 20, 35, 70	3 ^c^	3 ^d^	1	1	2 ^e^

^**a**^ each inoculum consisted of four different strains in equal concentration (8 Log10 cfu.mL^-1^)

^b^ not done at day 20

^c^ not done at days 13 and 35

^d^ not done at days 7 and 20

^e^ one microcosm analyzed at day 35 and at day 70

The ambient air temperature (AT) and relative humidity (RH) of the incubation room were measured and recorded at 5 min interval during the incubation period using a data logger (tinytagTGP-4500; Gemini Data Loggers Ltd, Chichester, UK) positioned at approximately 0.5 m above the bags. Ambient AT and RH averaged throughout the incubation period were 21.5 ± 1.4°C and 51.4 ± 8.1%, respectively.

Moisture content of the bedding was determined using gravimetric analysis. Bedding pH was measured from the entire content of each bag suspended in 20 mL deionized water using a pHM210 MeterLabTM (Radiometer Analytical, Hach Lange NZ, Auckland, New Zealand).

### Enumeration of *E*. *coli* O157:H7 and *S*. *enterica*

*E*. *coli* O157:H7 and *Salmonella* spp. were enumerated from the bedding microcosms using a three-tubes three-dilutions MPN method, which is a method suitable for enumeration of viable organisms in low concentration and in presence of particulate matter [30]. For enumeration of *E*. *coli* O157:H7, the entire content of the bedding microcosms were individually resuspended in 50 ml Modified Tryptic Soy Broth (mTSB; Fort Richard Laboratories) and mixed for 2 min in a Stomacher® 400 Circulator (Seward Limited, Worthing, United Kingdom). Serial dilutions of each suspended bedding were then prepared in mTSB, inoculated onto three 10 mL mTSB volume and incubated for 24h at 37°C followed by 24 h at 42°C. This primary enrichment was followed by selective plating on Cefixime tellurite sorbitol MacConkey agar (CT-SMAC; Fort Richard Laboratories) with plates incubated for 24 h at 42°C. The presence of *E*. *coli* O157:H7 on CT-SMAC plates from the three most appropriate dilutions of the initial suspension was confirmed by RT-PCR RapidFinder STEC screen and RapidFinder O157 confirmation Assays (Applied Biosystems™ by Thermo Fisher Scientific, Life Technologies Corporation, Austin, USA). A similar protocol was used to enumerate *S*. *enterica*; bedding suspension and serial dilutions were prepared in Buffered peptone water (BPW; Fort Richard Laboratories), primary enrichment was in BPW for 18 h at 35°C followed by Rappaport-Vassiliadis *Salmonella* enrichment medium (RVS; Fort Richard Laboratories) for 18 h at 42°C, and selective plating was on Xylose Lysine Deoxycholate Agar (XLD; Fort Richard Laboratories) for 18 h at 35°C. The presence of *Salmonella* spp. on XLD was confirmed by RT-PCR Salmonella confirmation Assay (Applied Biosystems™). The concentration of the bacterial target in the sample was determined by referencing the number of selective plates with confirmed bacterial growth to a three‐tube MPN probability table [31]. When the target was not detected (*i*.*e*., concentration below 4 (MPN).100 g^−1^), a value of 0.01 (MPN).g^-1^ (fresh weight) bedding was assigned to the MPN count. The concentrations of *E*. *coli* O157:H7 and *Salmonella* spp. were expressed as Logarithms to avoid overestimation and were reported as Log_10_ counts (MPN) g^-1^ (dry weight) bedding.

### Microbial count analysis

Statistical analysis of *E*. *coli* O157:H7 and *Salmonella* spp. Log_10_ counts were performed using two-way analysis of variance (ANOVA) in Genstat22 [32], with factors for pre-storage treatment and incubation time and the interaction term included. Fisher’s protected least significant differences at the 5% level (LSD) were used to compare the means of unstored and 2 months pre-storage groups (geometric means on the back-transformed scale) at each sampling time. Data from straw with and without manure were analyzed independently. Residual diagnostic plots were checked for departures from the assumptions of normality and constant variance.

### Bedding material data analysis

Mean moisture content and pH of the microcosm material was compared between unstored and pre-stored bedding over incubation time using a two-way analysis of variance (ANOVA), blocked by inocula (*E*. *coli* O157:H7, *S*. *enterica* or control), and fitted using Genstat22. The unstored and 2 months pre-storage means were compared at each incubation time using the LSD (5%). Data from straw with and without manure were analysed independently. Residual diagnostic plots were checked for departures from the assumptions of normality and constant variance.

### DNA Extraction and sequencing

To ensure thorough mixing, the frozen straw retrieved from -80°C storage was dried to constant weight at 105°C for a maximum of one hour and homogenized in a Waring blender (model 8011ES, Waring commercial, Torrington, Connecticut, USA). Total DNA was extracted from 0.1 g of the very finely blended material using a QIAamp Fast Stool Mini kit (Qiagen Inc., Mississauga, Canada) according to the manufacturer instructions. Illumina 16S V3-V4 libraries were prepared from the extracted DNAs and were sequenced using an Illumina MiSeq instrument using chemistry version3 (Massey Genome Services, Palmerston North, New Zealand).

### Bioinformatics methods

The processing of the amplicon reads followed a modified form of the pipeline described in [33]. The reads produced by the sequencing instrument were paired using the program FLASH2 [34]. Paired reads were then quality trimmed using Trimmomatic 0.38 [35]. The trimmed reads were then reformatted as fasta, and the read headers were modified to include the sample name. All reads were compiled in a single file, and the Mothur program suit [36] was used to remove reads with homopolymers longer than 10 nt, and then to collapse the reads into unique representatives. The collapsed reads were clustered with the program Swarm [37]. The clustered reads were filtered based on their abundance, keeping representatives that were a) present in one sample with a relative abundance >0.1%, b) present in >2% of the samples with a relative abundance >0.01% or c) present in 5% of the samples at any abundance level. The excluded OTUs (Operational Taxonomy Units) represented 6.5% of the reads, which suggested an acceptable quality of sequencing and data filtering. The selected OTUs were annotated using the Qiime program [38] with the Silva database v138 [39]. The annotated tables were then used for downstream statistical analysis. The data was loaded into R version 4.1.1 [40]. Proportions were calculated for each superkingdom set by dividing the counts of each taxon by the total sample counts. Principal coordinates analysis (PCoA) was performed with the “APE” package version 5.5 [41] on Bray-Curtis distances [42] calculated using the “vegan” R package version 2.5–7 [43]. Diversity estimates and ANOSIM [44] was also performed using the “vegan” R package with the default settings (including Bray-Curtis distance calculations). Permutation ANOVAs were performed using the lmPerm R package, version 2.1 [45] with 1 million permutations.

## Results

### Inactivation of *E*. *coli* O157:H7 and *S*. *enterica* in bedding materials over time

**Fig 1:**
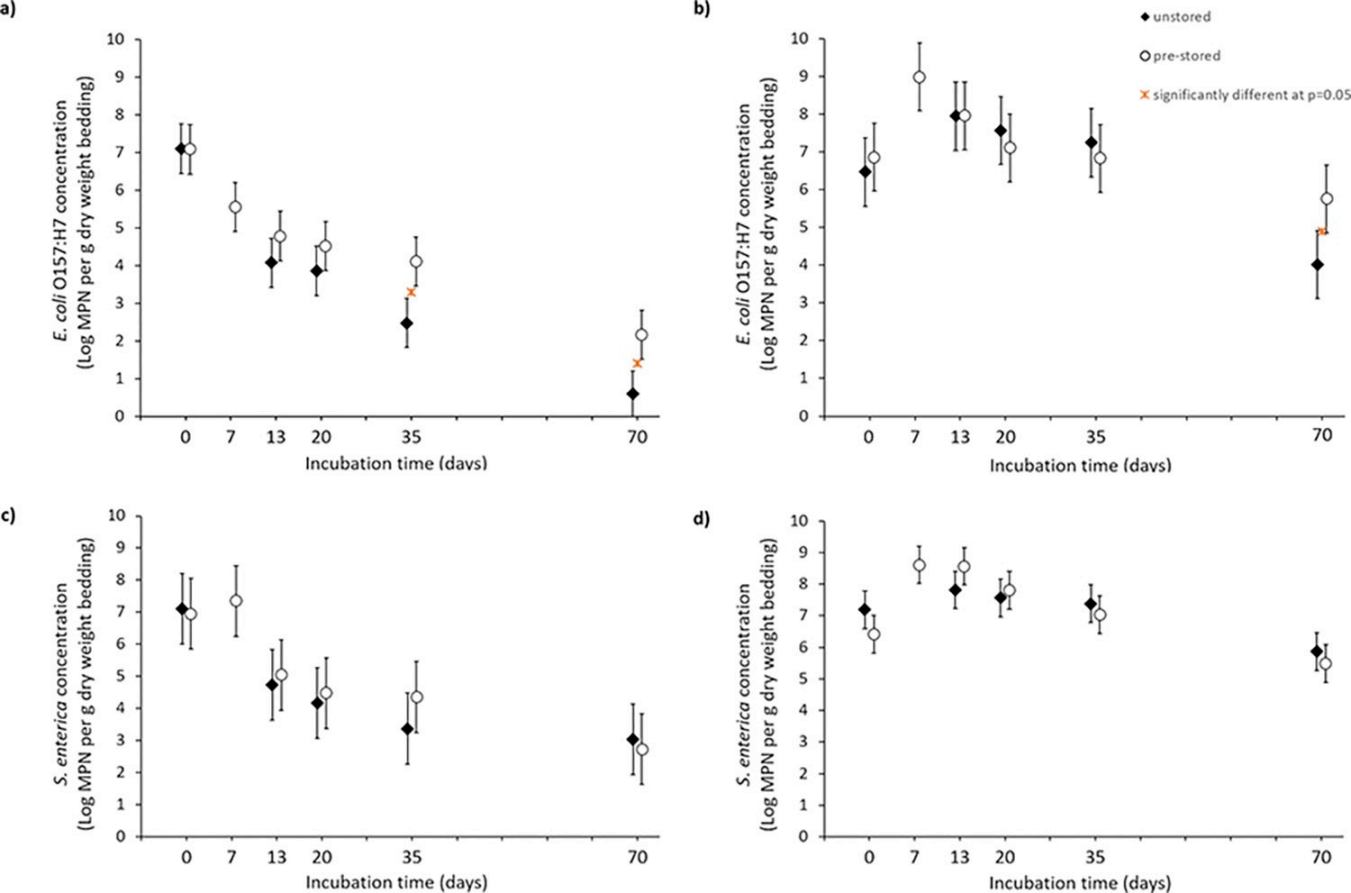
Inactivation of *E*. *coli* O157:H7 and *S*. *enterica* in unstored (filled diamond) and pre-stored (hollow circle) straw+manure bedding (a, c) and straw (b, d). Each MPN counts were obtained from triplicate bags. Bars are 95% confidence intervals for the geometric mean counts. * indicates significant difference at p = 0.05 between unstored and pre-stored geometric mean counts within an incubation time.

The numbers of introduced *E*. *coli* O157:H7 recovered immediately after introduction into the bedding microcosms were 7.1 Log_10_ g^-1^ (95% CI: 6.5–7.8) and 7.1 Log_10_ g^-1^ (95% CI: 6.4–7.7) in the unstored and prestored straw naturally contaminated with calf manure, respectively (Fig 1A), and 6.5 Log_10_ g^-1^ (95% CI: 5.6–7.4; unstored) and 6.9 Log_10_ g^-1^ (95% CI: 6.0–7.8; pre-stored) in the straw without calf manure (Fig 1B, **S1 Table**).

*E*. *coli* O157:H7 abundance in the straw naturally contaminated with calf manure changed significantly (p<0.01) over time, with a decreasing trend. The decrease was the greatest in the unstored bedding, with *E*. *coli* O157:H7 counts significantly (p<0.01) smaller than that in pre-stored bedding at 35 days of incubation. After 70 days incubation, the *E*. *coli* O157:H7 population in straw+manure was reduced by 6.6 Log_10_ g^-1^ in the unstored bedding (0.5 Log_10_ g^-1^ recovered; 95%CI: 0.0–1.2), and by 4.9 Log_10_ g^-1^ in the pre-stored bedding (2.2 Log_10_ g^-1^ recovered; 95%CI: 1.5–2.8). In straw without manure, the numbers of *E*. *coli* O157:H7 after 35-days incubation were similar to the initial counts. After 70 days, *E*. *coli* O157:H7 population was reduced by 2.5 Log_10_ g^-1^ in unstored straw, and by 1.1 Log_10_ g^-1^ in pre-stored straw, with a significant (p<0.05) difference between unstored and pre-stored straw. Naturally occurring *E*. *coli* O157:H7 were detected in a third of the control non inoculated microcosms (8/24 “straw+ manure”; 8/24 “straw” microcosms) but usually at low levels (<10 MPN.g^-1^ in in all eight *E*. *coli* O157-positive straw + manure microcosms and in 6/8 *E*. *coli* O157-positive straw microcosms).

The numbers of inoculated *S*. *enterica* recovered from the microcosms are presented in Fig 1C and 1d (and S1 Table). Immediately after inoculation, 7.1 Log_10_ g^-1^ (95%CI: 6.0–8.2), 7.0 Log_10_ g^-1^ (95%CI: 5.9–8.1), 7.2 Log_10_ g^-1^ (95%CI: 6.6–7.8) and 6.4 Log_10_ g^-1^ (95%CI: 5.8–7.0) were recovered from the unstored straw + manure, the pre-stored straw + manure, the unstored straw, and the pre-stored straw, respectively. In straw + manure, *S*. *enterica* decreased by 4.1 Log_10_ g^-1^ (unstored) and 4.3 Log_10_ g^-1^ (pre-stored) by incubation day 70, with no significant impact of pre-storage. In straw, *S*. *enterica* abundance remained for 35 days at levels comparable to or greater than the initial levels; by day 70, only a 1.3 (unstored) to 0.9 Log_10_ (pre-stored) g^-1^ reduction of *S*. *enterica* was achieved. Naturally occurring *Salmonella* spp. was recovered from half of the control microcosms (12/21 straw + manure; 9/21 straw) but at concentrations consistently <5 MPN.g^-1^

The mean moisture content of the bedding material was stable over the incubation time in both prestored and unstored straw + manure microcosms, while it declined (p<0.001) in the straw microcosms (Table 2, S1 Table). For the pre-stored straw + manure bedding, the mean pH values of bedding material were similar at day 0. By day 70, the mean pH of the pre-stored material was significantly (p<0.005) lower than that of the unstored material. For straw without manure, the mean pH increased over 70 days in both unstored straw and pre-stored material, with no difference in mean pH between stored and unstored straw at any time.

**Table 2:** Temporal changes of moisture content and pH of each bedding type over time.

Bedding Type		Moisture content (%) [mean (n = 3) % (95%CI)]		pH [mean (n = 3)(95%CI)]	
Material	Treatment	Day 0	Day 70	Day 0	Day 70
straw	unstored	51.8 (39.2–64.5)	33.0 (20.3–45.7)	7.0 (6.9–7.1)	8.9 (8.8–9.0)
	pre-stored	53.1 (40.4–65.8)	9.5 (0.0–22.1) *	6.9 (6.8–7.0)	8.7 (8.6–8.8)
Straw + manure	unstored	63.4 (51.0–75.8)	61.8 (49.4–74.2)	8.0 (7.8–8.2)	8.4 (8.2–8.6)
	pre-stored	69.8 (57.4–82.2)	67.7 (55.3–80.1)	8.1 (7.9–8.3)	7.1 (6.9–7.3)**

* Significant (p<0.001) difference between unstored and pre-stored bedding

** Significant (p<0.005) difference between unstored and pre-stored bedding

### Bacterial diversity and community profiles

Bacterial community profiling was carried out on each type of bedding after inoculation of the bacterial cocktail (day 0), and on days 35 and 70. A total of 1,0365,141 read pairs of 300 nt were obtained from the high-throughput sequencing of the 68 samples. The filtered reads were clustered into 718 species-level OTUs. Bacterial communities were reasonably characterized with the sampling effort for all bedding types as the rarefaction curves of the filtered OTUs approached horizontal (S1 Fig). More OTUs were represented in the straw + manure bedding in comparison to the straw bedding (p < 0.01) (S1 Fig).

Pre-storage of the bedding materials increased the number of bacterial taxa detected for each type of bedding; it significantly affected the richness of the microbial community (Chao1 (P = 0.01) and ACE (P = 0.01)), but not its eveness (Shannon (P = 0.65) and Simpson (P = 0.82) indexes) (Table 3).

**Table 3:** Alpha-diversity indices of the bacterial population detected in two bedding materials (“straw” and “straw + manure”) with and without pre-storage treatment. Values represent average ± standard deviation.

Index	Straw		Straw + manure		Permutation ANOVA p-value	
	Unstored (n = 16)	Pre-stored (n = 16)	Unstored (n = 18)	Pre-stored (n = 18)	Storage treatment	Presence of manure
Chao1	216.38 ± 9.91	244.89 ± 9.6	506.29 ± 4.09	520.53 ± 9.12	0.011	< 0.0001
ACE	212.72 ± 8.97	237.09 ± 8.63	501.42 ± 3.76	515.03 ± 8.81	0.012	< 0.0001
Shannon	2.21 ± 0.16	2.11 ± 0.12	4.31 ± 0.03	4.34 ± 0.03	0.654	< 0.0001
Simpson	0.73 ± 0.05	0.71 ± 0.04	0.97 ± 0	0.97 ± 0	0.823	< 0.0001

**Fig 2:**
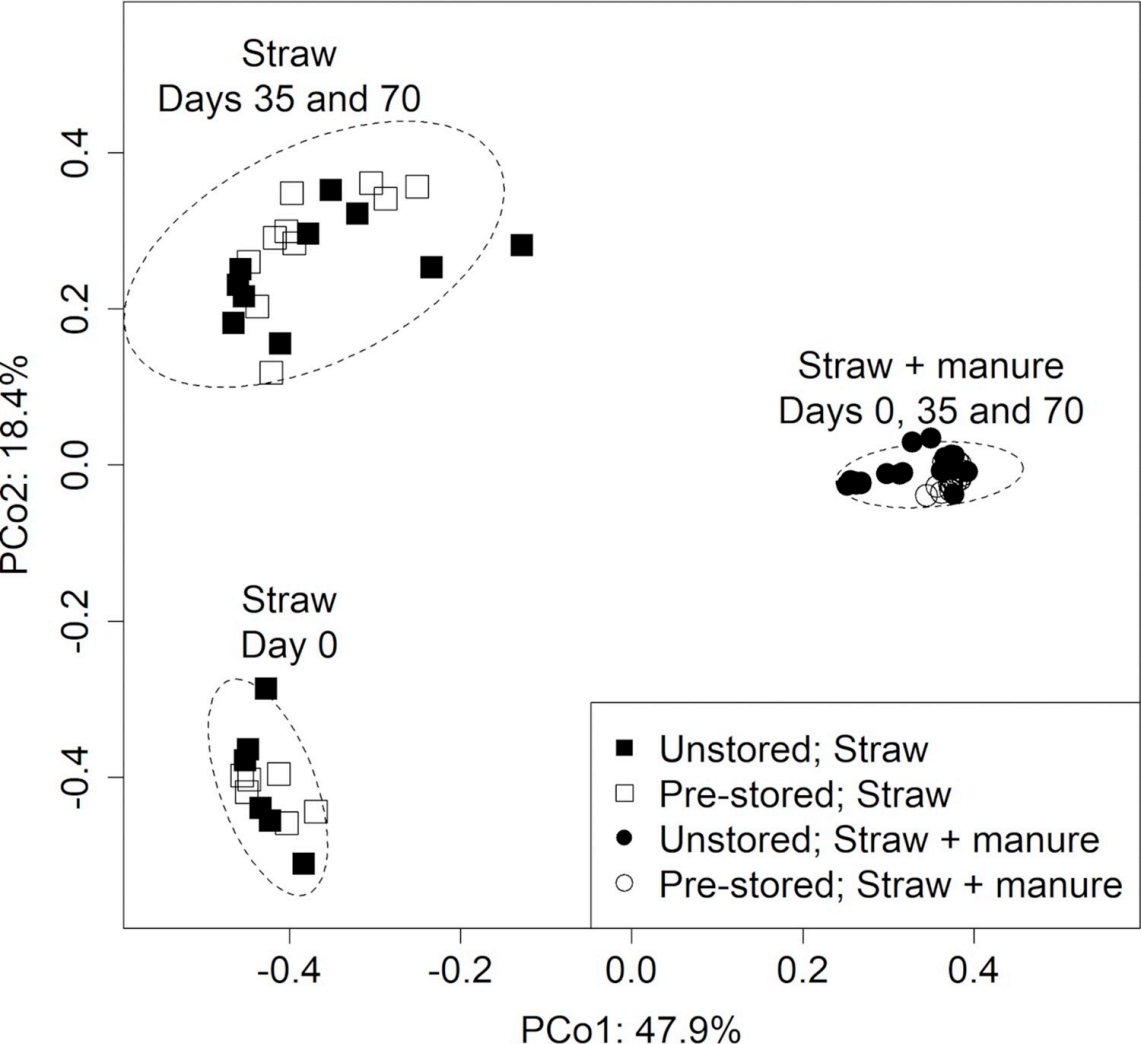
**Bray-Curtis principal coordinate analysis (PCoA) illustrating the changes in bacterial community beta-diversity in the four beddings types over incubation time.** Each symbol represents a sample, with distance between samples calculated using Bray-Curtis similarity measures. Dashed ellipses indicate 95% confidence intervals of the ordinations for named clusters.

Principal coordinates analyses (PCoA) based on Bray-Curtis indice revealed the bacterial communities in straw and straw + manure were significantly different (R = 0.46316, p<0.0001; perm = 1,000 analysis of variance using distance matrices) (Fig 2). In straw microcosms, a shift in the bacterial community was detected between days 0 and 35 in both pre-stored and unstored straw. Comparatively, the bacterial community in the straw + manure exhibited a more consistent diversity over time. The bacterial communities in straw + manure clustered by pre-storage at day 0, but not at day 35 and day 70. Neither the bacterial communities of the straw or that of the straw + manure were affected by the inoculation with *E*. *coli* O157:H7 or *S*. *enterica*.

### Taxonomic composition of bacterial communities

**Fig 3:**
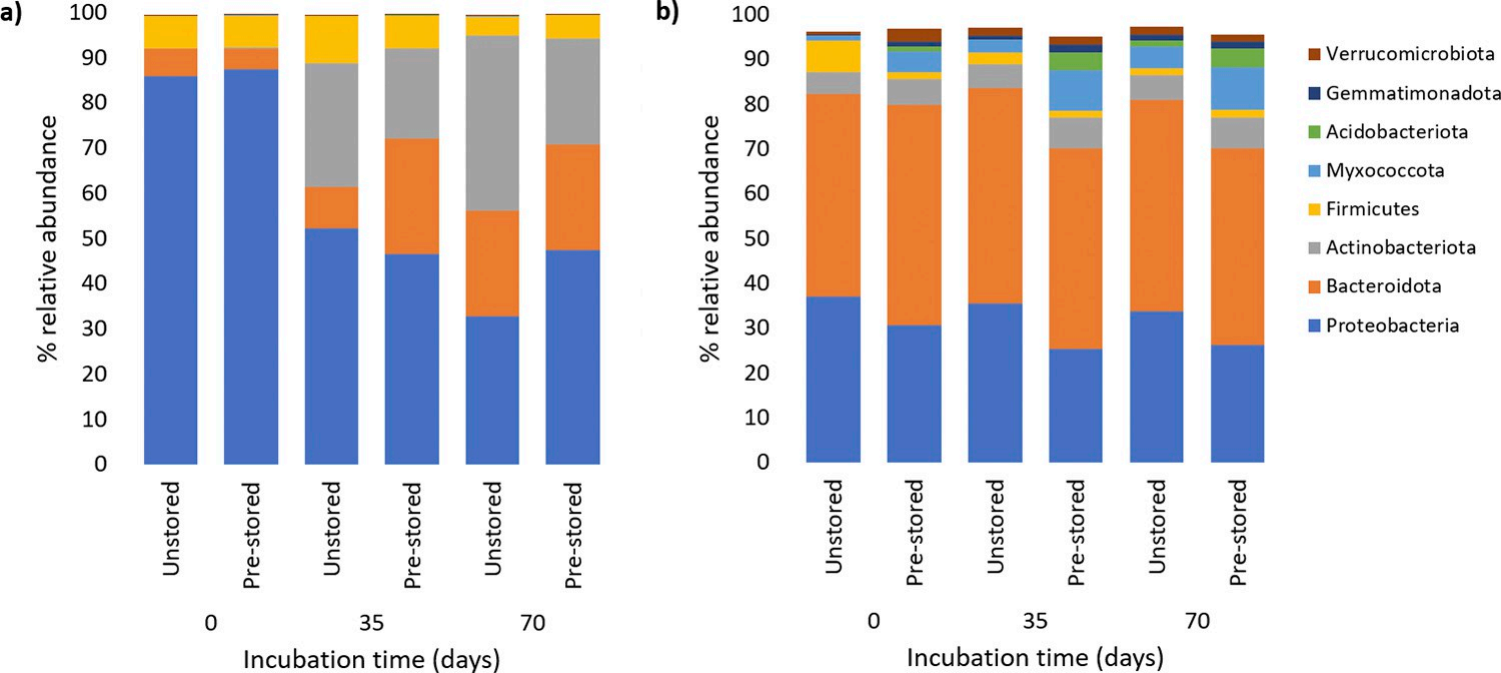
**Change of relative abundance of major bacterial phyla across bedding types (a: straw; b: straw + manure) with and without pre-storage treatment and over incubation time.** Only the phyla with abundance above 1.0% were plotted. Bars are standard error of mean (n = 6).

At the phylum level and regardless of the pre-storage treatment, the bacterial communities of the straw bedding on day 0 were largely dominated by Proteobacteria (unstored: 85.6%; pre-stored: 87.6%; p = 0.641), followed by Firmicutes (unstored: 7.2%; pre-stored: 7.0%; p = 0.131), Bacteroidota (pre-stored: 4.5%; unstored: 6.1%; p = 0.722) and Actinobacteriota (pre-stored: 0.3%; unstored: 0.1%; p = 0.433)(Fig 3 and S1 Table).

Proteobacteria abundance was reduced significantly over time (p-values of <0.001 and 0.0016), with observed abundance of 52.4% and 46.7% on day 35 and 32.9% and 47.2% on day 70 in unstored straw or pre-stored straw, respectively. The Firmicute abundance in straw was reduced over time, with no significant difference between pre-stored and unstored bedding on day 70 (unstored: 4.4%; pre-stored: 5.32; p = 0.2987). In contrast to Proteobacteria and Firmicutes phyla, the Bacteriodota abundance in straw bedding increased over time to reach an average abundance of 23% on day 70, with significant difference between unstored and pre-stored straw on day 35 (pre-stored: 25.4%; unstored: 9.7%; p<0.0001). Actinobacteriota abundance in straw increased significantly (p<0.0001) over time despite the large inter-sample variability observed on day 35 (pre-stored: 10.98–34.21%; unstored: 12.4–43.2%) and on day 70 (pre-stored:10.16–34.0%; unstored: 7.6–64.15%).

In straw + manure bedding, the bacterial communities on day 0 mainly consisted of Bacteroidota (pre-stored straw, 49%; unstored straw, 45% p = 0.1547) and Proteobacteria (pre-stored straw, 37%; unstored straw, 30%; p = 0.1072), followed by Actinobacteriota (pre-stored straw, 5%; unstored straw, 5%; p = 0.6844) and Firmicutes (pre-stored straw, 1.6%; unstored straw, 3% p = 0.571). Additional bacterial phyla represented in straw + manure microcosms included Myxococcota (pre-stored, 4.6%; unstored, 1.2%; p<0.0001), and Verrucomicrobiota, Desulfobacterota, Bdellovibrionota, Fibrobacterota, Acidobacteriota and Spirochaetota, which were each detected in abundance of 1–3%. The bacterial community in straw + manure was still dominated by Bacteriodota and Proteobacteria on day 70, although the relative abundance of each phylum was affected by time and pre-storage treatment: Bacteroidota was significantly more represented in unstored manure + straw than in pre-stored bedding on day 35 (pre-stored, 44.87%; unstored, 48.08%; p = 0.0002) and on day 70 (pre-stored, 43.79%; unstored, 47.09%; p<0.0001, respectively). Similarly, Proteobacteria were more abundant in the unstored straw + manure bedding compared to the pre-stored bedding on both day 35 (pre-stored, 25%; unstored, 35%; p<0.0001) and day 70 (pre-stored, 26%; unstored, 33%; p = 0.0127). In contrast, Actinobacteriota were significantly less represented on day 70 in unstored straw + manure than in the pre-stored straw + manure (pre-stored, 7.0%; unstored, 5.4%; p = 0.0075). Myxococcota, Acidobacteriota and Verrucomicrobiota were also less represented in the unstored straw + manure bedding on day 70 compared to the pre-stored bedding.

At the order level, the most abundant Proteobacteria orders that were identified were Xanthomonadales (27.5%) and Pseudomonadales (4.8%) in straw; Cellvibrionales (7.6%), Burkholderiales (4.5%) and Xanthomonadales (10.6%) in straw+manure. The most common order within the Bacteriodota were Flavobacteriales (*Moheibacter sp*., *Muricauda spp*., *Sinomicrobium spp*.) (23.7% in straw+manure, 8.1% in straw) and Sphingobacteriales (*Parapedobacter* spp.) (8.1% in straw+manure, 5.2% in straw), in both straw+manure and straw. Interestingly, the abundance of the Sphingobacteriales order seemed to be consistent with *E*. *coli* O157:H7 inactivation rate: Sphingobacteriales were less abundant in straw compared to straw+manure, and were more abundant in the unstored straw + manure than in stored straw + manure on days 35 and day 70. The most common Firmicutes orders were Bacillales *(Bacillus sp*.) (3.9%) and Staphylocaccaceae (0.2%) in straw. The most common Actinobacteriota genus were *Nocardiopsis* spp. (11.5%) in straw and *Nocardiopsis* spp. (0.3%) *and Glycomyces* spp. (1.5%) in straw + manure.

## Discussion

The study explored the effectiveness of straw animal beddings to inactivate *E*. *coli* O157:H7 and *S*. *enterica* introduced in the beddings after different storage time, and the possible role of the indigenous bacterial community. Results revealed that pre-storage of the bedding materials (for approx. 2 months prior to pathogen inoculation) was associated with a slower inactivation of *E*. *coli* O157:H7 (but not of *S*. *enterica*), and affected the composition but not the diversity of the indigenous bacterial community.

The two types of bedding materials (straw prior to use, or straw + manure) were sourced from a dairy farm at the end of the calving season. A cocktail of either four strains of *E*. *coli* O157:H7 or four strains of *S*. *enterica* was introduced in the bedding microcosms in order to include a possible strain-to-strain difference effect and determine the upper tolerance to inactivation; the high inoculum level simulated the worst-case pathogen loads possibly contaminating a bedding pile. Cattle, particularly calves and heifers, can excrete multiple genotypes of either *E*. *coli* O157:H7 or *Salmonella* spp., and at levels ranging from 10^2^ to 10^7^ CFU g^−1^ in feces [46–49].

The *E*. *coli* O157:H7 and *S*. *enterica* introduced into the experimental microcosms survived for the 10-week duration of the experiment, however *E*. *coli* O157:H7 numbers, and to a lesser extent *Salmonella spp*. numbers, declined over time. These findings were similar with those previously reported for abattoir wastes, bovine feces, or manure amended soils [23, 50, 51], where, in general terms, the population size of pathogenic bacteria was shown to progressively decline. Many studies that investigated persistence *E*. *coli* O157:H7 in different soils noted the importance of indigenous microbial diversity on the persistence of this pathogen. For example, Jiang et al. [51] and Baker et al. [52], who experimentally manipulated microbial diversity by sterilization and/or dilution-re-inoculation approaches, observed a greater survival of *E*. *coli* O157:H7 in autoclaved soils in comparison to unautoclaved soils. Survival time in autoclaved soils decreased with manure amendment [51, 53]. A more rapid decline of *E*. *coli* O157:H7 was reported in soils with an increasing history of solid manure addition [26]. *E*. *coli* O157:H7 persistence was strongly and negatively correlated with indigenous bacterial richness [52, 53]. Similarly, the decline of *S*. *enterica* was found to be more rapid in soils amended with cover crops and compost that had higher bacteria diversity compared to the same soils treated with synthetic fertilizers and had a low bacterial diversity [54]. Extrapolating the fate of zoonotic bacteria in soils to animal beddings needs to be done with caution, as there are many parameters that can affect microbial community -including C/N ratio, bulk density, water holding capacity, porosity or average particle size- that differ among soil, manure and straw-bedding [55]. In the present study, the greatest decline of *E*. *coli* O157:H7 or *S*. *enterica* was observed when the bacterial community was highly diverse (e.g., in straw + manure), which is consistent with observations in soils. This indicates the negative impact of a diverse indigenous bacterial community on the persistence of zoonotic bacteria in animal bedding. Our study showed moreover that the inactivation of *E*. *coli* O157:H7 was slower when the bacteria were introduced into pre-stored bedding, confirming a previous observation in wastewater biosolids that the antagonistic effect of the indigenous bacterial community towards introduced food-borne pathogens declined with length of biosolids storage [56]. Several mechanisms, including competition for resources, direct antagonism, or predation, have been proposed to explain the role of the resident bacterial community [54, 57].

Habitats high in macronutrients or organic matter tend to contain more diverse bacterial communities and can positively affect suppression of foodborne pathogen through a greater use of resources [54]. The survival of Shiga toxin-producing *E*. *coli* has been related to the number of carbon sources used by the resident microbial communities. This is because the higher the number of carbon sources used by the microbial communities, the lower the number that will be available for utilization by introduced *E*. *coli* strains [53]. Ecosystems yielding a higher level of biodiversity are also in general less vulnerable to disturbances than ecosystems that have a lower diversity [58]. Abundant interactions within a microbial community were shown to result in suppression of colonization by an invader *E*. *coli* O157:H7 [57, 59]. Our study found a greater initial shift in the bacterial community composition in straw compared to straw + manure, in which inactivation of the added pathogens appeared to be more effective. It is possible that the diverse bacterial community in the bedding containing manure was more stable to the perturbation associated with inoculation with the food-borne pathogens, and as a result more able to resist invasion. It is also possible that the *E*. *coli* O157:H7 or *Salmonella* were better able to survive when the bacterial community was “unstable” or less able to resist a perturbation, as was apparently the case for straw without manure. Further work is needed to better understand the biological mechanisms that result in inactivation of the introduced pathogens in perturbed ecosystems.

In the present study, the inactivation of both *E*. *coli* O157:H7 and *S*. *enterica* was concomitant to a reduction in Proteobacteria abundance. This finding differs from the negative correlation between survival of *E*. *coli* O157:H7 and Proteobacteria abundance established in soils and sand-based beddings [53, 60]. However, the influence of the different members within this phylum was shown to vary. For example, bacteria belonging to Beta-Proteobacteria order in particular have shown a suppressive effect on *E*. *coli* O157:H7, while other orders were thought to enhance survival [60]. The efficiency of resource utilization by the “invader” was proposed to be the main driver, at least when considering species interactions on the basis of one resource at a time [61]. In our study, close observation of the bacterial orders within the Proteobacteria showed dominance by Gamma-proteobacteria, such as Cellvibrionales, Burkholderiales and Xanthomonadales in straw+manure beddings, and by Xanthomonadales in straw. As these orders are known to degrade high-molecular weight or complex plant-based compounds, including cellulose, xylan, or starch [62], our study may indicate synergy between Proteobacteria and *E*. *coli* O157:H7 through use of available nutrient, rather than competition by antagonism.

The high abundance of Bacteroides observed when *E*. *coli* O157:H7 and *Salmonella* spp. are inactivated is in agreement with previous research in sand-based bedding and in soils that showed that the Bacteroides phylum as a whole exhibited a negative correlation with survival of *E*. *coli* O157:H7, with some specific species displaying a particularly suppressive effect [8, 60]. This is consistent with our finding that the relative abundance of the Sphingomonadales order was greater in prestored straw bedding where the inactivation of *E*. *coli* O157:H7 was slower, comparative to inactivation in unstored bedding. Bacteroidetes are known for their ability to initiate the degradation of complex [63], suggesting that the suppressive effect of Bacteriodes was possibly attributed to a more efficient use of complex lignocellulolytic resources.

Actinobacteria are present in a wide range of ecosystems and have a pivotal role in plant residues degradation, particularly in the less fertile environments [64, 65]. While the survival of zoonotic bacteria in soil has been found to be positively affected by Actinobacteria [60], some members of the Actinobacteria, including Nocardiopsis species, are also known to produce an array of bioactive compounds active against *E*. *coli* [66]. In the present study, the comparison between stored and not stored beddings confirms that storage of straw + manure bedding might have affected nutrient availability but did not reveal a synergist or suppressive effect of Actinobacteria against *Salmonella* or *E*. *coli* O157:H7.

Compared to un-stored beddings, pre-stored bedding contained a greater number of bacterial taxa in relatively low abundance and that did not significantly affect the community structure. Examples include bacteria of the phyla Bdellovibrionota and Myxococcota, which have demonstrated an ability to enter into and lyse *E*. *coli* or *Salmonella* cells [67–69]. However, the slower inactivation of *E*. *coli* O157:H7 in pre-stored beddings suggests that the increase in abundance of these bacterial phyla was unlikely to result in these phyla being present in number large enough to significantly affect zoonotic inactivation. Reasons for this could be a different relative location of target and low abundant predatory bacterial cells within the bedding material, or a specific physiological inability to parasitise *E*. *coli* O157:H7 or *S*. *enterica* in our experimental conditions.

As a general observation, the abundance of bacterial orders known to adapt to “dry” “salty” conditions increased over time on both beddings and was also greater in stored bedding compared to bedding that had not been stored. This suggests the possibility that salt levels increased during storage due to evaporation [54], which is in general agreement with the reduction of water content in stored beddings. In the current study, *S*. *enterica* tended to be inactivated less effectively than *E*. *coli* O157:H7 in both pre-stored bedding types, corroborating previous findings that *Salmonella* spp. better resists desiccation and other various environmental stresses than others zoonotic *Enterobacteriaceae* [70, 71]. The effect of water content alone on inactivation of *Salmonella* spp or *E*. *coli* O157:H7 in our study remains unclear. For instance, the longer survival of *E*. *coli* O157:H7observed on straw, which had a drier content than straw + manure, as well as the difference in inactivation between unstored and pre-stored straw + manure material despite their similar water content, suggests that the effect of water content on inactivation likely involves complex mechanisms. A similar unclear effect of water content on survival of *E*. *coli* O157:H7 was also reported in manure piles, with studies reporting a more frequent recovery of the bacteria from the middle and bottom of unaerated manure piles than from the top, and others reporting a longer survival time of pathogenic *E*. *coli* on the outside compared to the inside of the piles [21, 72]. Further studies considering the interaction effects between bedding-water content and nutrients availability or microflora population are required to better assess the effect of water content on bacterial inactivation and to develop recommandation for bedding management.

pH has been repeatedly shown to exert a critical role on the inactivation of *E*. *coli* O157:H7 and *S*. *enterica* in agricultural soils [24]. In the current study, the inactivation of *E*. *coli* O157:H7 and *S*. *enterica* did not seem to be reflected by the pH changes in the neutral to alkaline gradient. We observed that higher pH values could produce longer survival time of *E*. *coli* O157:H7 strains, but also that, at high pH conditions, survival times were shorter when diversity of the bacterial community was high. It is likely that the effect of pH on survival time of *E*. *coli* O157:H7 in bedding was modulated by the bacterial community, as previously proposed for soil ecosystems [53]. The increase in Bacteriodota and Actinobacteriota relative abundances in beddings with alkaline pH, which was expected given previous reports [73, 74], suggests a particular link between these phyla, pH and *E*. *coli* O157:H7 or *Salmonella spp*. inactivation. Further investigation in animal beddings would be needed to determine the causal relationship between pH gradient, the presence and role of bacterial taxa and the inactivation of *E*. *coli* 157:H7 or *Salmonella spp*.

Care was taken at each step of our experiment to reduce the biological variability inherent to pastoral complex environments, and as a result, our experimental microcosms and pre-storage conditions did not fully reflect those in field or large-scale bedding heaps. In order to better guide effective bedding management practices or assess risks associated with the storage and recycling of animal beddings sourced from dairy farms, future studies should consider the inactivation of zoonotic bacteria when introduced in animal beddings at a low concentration, in association with organic matter, in different growth phase, or in different types of bedding substrates. Establishing a correlation between inactivation of zoonotic bacteria in pre-storage conditions representative of a range of farm practices and phylogenic data would also be critical to confirm key OTUs involved in lowering the levels of zoonotic bacteria.

In summary, this research contributed to the fundamental understanding of the fate of zoonotic bacteria introduced in animal beddings during storage and identified that bedding material storage conditions pre-and post use in animal facilities could be important to prevent the risk of zoonosis dissemination to the environment or to the dairy herds. For instance, slower inactivation of zoonotic bacteria in pre-stored beddings suggests that continuous addition of fresh manure or bedding on a stock pile would increase the risk of remaining zoonotic pathogens, and that a quarantine period during which no new manure or used bedding is added to the manure heaps should be observed before application on agricultural land. Sporadic detection of the two pathogens at low concentration in the straw material prior to use in the animal facility suggests a point contamination, such as wildlife excreta. Birds and rodents can contribute to the cycling of food-borne bacteria on farm [75], and implementation of biosecurity measures targetting wildlife around the storage areas of bedding material prior to use in animal facilities should be encouraged to control the risks of recontaminating the animals or introducing new strains in the herds. In terms of maturation practices, our findings suggest that storage of used animal beddings under conditions promoting a diverse and stable bacterial community might improve zoonosis inactivation. Overall, development of guidelines on bedding storage management pre and post use in animal facilities and impact on pathogens inactivation are recommended.

## Supporting information

S1 Fig Rarefaction curves showing bacterial community richness in straw (black) and straw+manure (red) beddings. (TIFF)

S1 Table Bacterial concentration, pH and humidity, and relative abundance of bacterial phyla in the microcosms. (XLSX)

## References

[1] OECD/FAO. OECD-FAO Agricultural Outlook 2021–2030. Paris: OECD Publishing; 2021. p. 337.

[2] Ferreira Ponciano Ferraz P, Araújo e Silva Ferraz G, Leso L, Klopčič M, Rossi G, Barbari M. Evaluation of the Physical Properties of Bedding Materials for Dairy Cattle Using Fuzzy Clustering Analysis. Animals. 2020;10(2):351. doi: 10.3390/ani10020351 32098358 PMC7070853

[3] Sutherland MA, Worth GM, Cameron C, Ross CM, Rapp D. Health, physiology, and behavior of dairy calves reared on 4 different substrates. Journal of Dairy Science. 2017;100(3):2148–56. doi: 10.3168/jds.2016-12074 28109608

[4] Tucker CB, Weary DM, von Keyserlingk MAG, Beauchemin KA. Cow comfort in tie-stalls: Increased depth of shavings or straw bedding increases lying time. Journal of Dairy Science. 2009;92(6):2684–90. doi: 10.3168/jds.2008-1926 19448001

[5] Vida C, de Vicente A, Cazorla FM. The role of organic amendments to soil for crop protection: Induction of suppression of soilborne pathogens. Annals of Applied Biology. 2020;176(1):1–15. doi: 10.1111/aab.12555

[6] Pell AN. Manure and Microbes: Public and Animal Health Problem? Journal of Dairy Science. 1997;80(10):2673–81. doi: 10.3168/jds.S0022-0302(97)76227-1 9361239 PMC7130904

[7] Beauchemin J, Fréchette A, Thériault W, Dufour S, Fravalo P, Thibodeau A. Comparison of microbiota of recycled manure solids and straw bedding used in dairy farms in eastern Canada. Journal of Dairy Science. 2022;105(1):389–408. doi: 10.3168/jds.2021-20523 34656347

[8] Westphal A, Williams ML, Baysal-Gurel F, LeJeune JT, Gardener BBM. General Suppression of *Escherichia coli* O157:H7 in Sand-Based Dairy Livestock Bedding. Applied and Environmental Microbiology. 2011;77(6):2113–21. doi: 10.1128/AEM.01655-10 21257815 PMC3067323

[9] Rapp D, Ross CM, Maclean P, Cave VM, Brightwell G. Investigation of On-Farm Transmission Routes for Contamination of Dairy Cows with Top 7 *Escherichia coli* O-Serogroups. Microbial Ecology 2021;81(1):67–77. doi: 10.1007/s00248-020-01542-5 32561945

[10] EFSA and ECDC (European Food Safety Authority and European Centre for Disease Prevention and Control). The European Union One Health 2020 Zoonoses Report. European Food Safety Authority Journal. 2021;19(e6971):324. doi: 10.2903/j.efsa.2021.6971 36329690 PMC9624447

[11] Omer MK, Álvarez-Ordoñez A, Prieto M, Skjerve E, Asehun T, Alvseike OA. A Systematic Review of Bacterial Foodborne Outbreaks Related to Red Meat and Meat Products. Foodborne Pathogens and Diseases. 2018;15(10):598–611. doi: 10.1089/fpd.2017.2393 29957085

[12] U.S. Food and Drug Administration. Factors potentially contributing to the contamination of romaine lettuce implicated in the three outbreaks of *E*. *coli* O157:H7 during the fall of 2019 2020. Available from: https://www.fda.gov/food/outbreaks-foodborne-illness/factors-potentially-contributing-contamination-romaine-lettuce-implicated-three-outbreaks-e-coli. Accessed 15Feb2023.

[13] Centers for Disease Control and Prevention (CDC). Outbreaks of *Escherichia coli* O157:H7 associated with petting zoos-North Carolina, Florida, and Arizona, 2004 and 2005. Morbidity and Mortality Weekly Report. 2005;54:1277–80. 16371942

[14] Gu G, Strawn LK, Oryang DO, Zheng J, Reed EA, Ottesen AR, et al. Agricultural Practices Influence Salmonella Contamination and Survival in Pre-harvest Tomato Production. Frontiers in Microbiology. 2018;9. doi: 10.3389/fmicb.2018.02451 30386314 PMC6198144

[15] U.S. Food and Drug Administration. FDA Fact sheet. Produce Safety Rule (21 CFR 112): Biological soil amendments of animal origin. 2015. p. 3.

[16] Larney FJ, Buckley KE, Hao X, McCaughey WP. Fresh, Stockpiled, and Composted Beef Cattle Feedlot Manure. Journal of Environmental Quality. 2006;35(5):1844–54. doi: 10.2134/jeq2005.0440 16899756

[17] Morse Meyer D, Garnett I, Guthrie JC. A Survey of Dairy Manure Management Practices in California. Journal of Dairy Science. 1997;80(8):1841–5. doi: 10.3168/jds.S0022-0302(97)76119-8

[18] Hubbe MA, Nazhad M, Sánchez C. Composting as a way to convert cellulosic biomass and organic waste into high-value soil amendments: A review. BioResources. 2010;5(4):2808–54. doi: 10.15376/biores.5.4.2808–2854

[19] Lasprilla-Mantilla MI, Wagner V, Pena J, Frechette A, Thivierge K, Dufour S, et al. Effects of recycled manure solids bedding on the spread of gastrointestinal parasites in the environment of dairies and milk. Journal of Dairy Science. 2019;102(12):11308–16. doi: 10.3168/jds.2019-16866 31548050

[20] Duan H, Ji M, Xie Y, Shi J, Liu L, Zhang B, et al. Exploring the Microbial Dynamics of Organic Matter Degradation and Humification during Co-Composting of Cow Manure and Bedding Material Waste. Sustainability. 2021;13(23). doi: 10.3390/su132313035

[21] Sun L, Han X, Li J, Zhao Z, Liu Y, Xi Q, et al. Microbial Community and Its Association With Physicochemical Factors During Compost Bedding for Dairy Cows. Frontiers in Microbiology. 2020;11. doi: 10.3389/fmicb.2020.00254 32153538 PMC7047772

[22] Hutchison ML, Walters LD, Avery SM, Moore A. Decline of zoonotic agents in livestock waste and bedding heaps. Journal of Applied Microbiology. 2005;99(2):354–62. doi: 10.1111/j.1365-2672.2005.02591.x 16033467

[23] Kudva IT, Blanch K, Hovde CJ. Analysis of *Escherichia coli* O157:H7 Survival in Ovine or Bovine Manure and Manure Slurry. Applied and Environmental Microbiology. 1998;64(9):3166–74. doi: 10.1128/AEM.64.9.3166–3174.1998 9726855 PMC106705

[24] Ongeng D, Geeraerd AH, Springael D, Ryckeboer J, Muyanja C, Mauriello G. Fate of *Escherichia coli* O157:H7 and *Salmonella enterica* in the manure-amended soil-plant ecosystem of fresh vegetable crops: A review. Critical Reviews in Microbiology. 2015;41(3):273–94. doi: 10.3109/1040841X.2013.829415 24083946

[25] You Y, Rankin SC, Aceto HW, Benson CE, Toth JD, Dou Z. Survival of *Salmonella enterica* Serovar Newport in Manure and Manure-Amended Soils. Applied and Environmental Microbiology. 2006;72(9):5777–83. doi: 10.1128/AEM.00791-06 16957193 PMC1563654

[26] Franz E, Semenov AV, Termorshuizen AJ, De Vos OJ, Bokhorst JG, Van Bruggen AHC. Manure-amended soil characteristics affecting the survival of *E*.* coli* O157:H7 in 36 Dutch soils. Environmental Microbiology. 2008;10(2):313–27. doi: 10.1111/j.1462-2920.2007.01453.x 18199123

[27] Semenov AV, van Overbeek L, Termorshuizen AJ, van Bruggen AHC. Influence of aerobic and anaerobic conditions on survival of *Escherichia coli* O157:H7 and *Salmonella enterica* serovar Typhimurium in Luria–Bertani broth, farm-yard manure and slurry. Journal of Environmental Management. 2011;92(3):780–7. doi: 10.1016/j.jenvman.2010.10.031 21035246

[28] Ross CM, Rapp D, Cave VM, Brightwell G. Prevalence of Shiga toxin-producing *Escherichia coli* in pasture-based dairy herds. Letters in Applied Microbiology. 2019;68(2):112–9. doi: 10.1111/lam.13096 30411807

[29] Axmann H, Sebastianelli A, Arrillaga JL. Sample preparation techniques of biological material for isotope analysis. Seibersdorf, Austria. International Atomic Energy Agency (IAEA). 1990.

[30] Blodgett R. BAM Appendix 2: Most Probable Number from Serial Dilutions. In FDA’s Bacteriological Analytical Manual (BAM). U.S. Food and Drug Administration, editor. 2010. Available from: https://www.fda.gov/food/laboratory-methods-food/bam-appendix-2-most-probable-number-serial-dilutions. Accessed 15 Feb2023.

[31] World Health Organization. Guidelines for Drinking‐Water Quality, Vol. 1: Recommendations. WHO document production services, Editor. Geneva: 1984. p. 115.

[32] GenStat. GenStat® for Windows^TM^, 22^nd^ Edition. VSN International, Hemel Hempstead. 2022. Available from: URL Genstat.co.uk.

[33] Camarinha-Silva A, Jáuregui R, Pieper DH, Wos-Oxley ML. The temporal dynamics of bacterial communities across human anterior nares. Environmental Microbiology Reports. 2012;4(1):126–32. doi: 10.1111/j.1758-2229.2011.00313.x 23757239

[34] Magoč T, Salzberg SL. FLASH: fast length adjustment of short reads to improve genome assemblies. Bioinformatics. 2011;27(21):2957–63. doi: 10.1093/bioinformatics/btr507 21903629 PMC3198573

[35] Bolger AM, Lohse M, Usadel B. Trimmomatic: a flexible trimmer for Illumina sequence data. Bioinformatics. 2014;30(15):2114–20. doi: 10.1093/bioinformatics/btu170 24695404 PMC4103590

[36] Schloss PD, Westcott SL, Ryabin T, Hall JR, Hartmann M, Hollister EB, et al. Introducing mothur: Open-Source, Platform-Independent, Community-Supported Software for Describing and Comparing Microbial Communities. Applied and Environmental Microbiology. 2009;75(23):7537–41. doi: 10.1128/AEM.01541-09 19801464 PMC2786419

[37] Mahé F, Rognes T, Quince C, de Vargas C, Dunthorn M. Swarm: robust and fast clustering method for amplicon-based studies. PeerJ. 2014;2(e593). doi: 10.7717/peerj.593 25276506 PMC4178461

[38] Caporaso JG, Kuczynski J, Stombaugh J, Bittinger K, Bushman FD, Costello EK, et al. QIIME allows analysis of high-throughput community sequencing data. Nature Methods. 2010;7(5):335–6. doi: 10.1038/nmeth.f.303 20383131 PMC3156573

[39] Quast C, Pruesse E, Yilmaz P, Gerken J, Schweer T, Yarza P, et al. The SILVA ribosomal RNA gene database project: improved data processing and web-based tools. Nucleic Acids Research. 2013;41:D590–6. doi: 10.1093/nar/gks1219 23193283 PMC3531112

[40] Team RC. A language and environment for statistical computing. Vienna, Austria.: R Foundation for Statistical Computing; 2021. Available from: URL https://www.R-project.org/.

[41] Paradis E, Schliep K. Ape 5.0: an environment for modern phylogenetics and evolutionary analyses in R. Bioinformatics. 2018;35(3):526–8. doi: 10.1093/bioinformatics/bty633 30016406

[42] Bray JH, Maxwell SE. Multivariate analysis of variance SAGE Publications, Inc; 1985. 80 p.

[43] Dixon P. VEGAN, a package of R functions for community ecology. Journal of Vegetation Science. 2003;14(6):927–30. doi: 10.1111/j.1654-1103.2003.tb02228.x

[44] Clarke KR. Non-parametric multivariate analyses of changes in community structure. Australian Journal of Ecology. 1993;18(1):117–43. doi: 10.1111/j.1442-9993.1993.tb00438.x

[45] Wheeler B, Torchiano M. Permutation Tests for Linear Models 2016. Available from: https://github.com/mtorchiano/lmPerm.

[46] Rhoades JR, Duffy G, Koutsoumanis K. Prevalence and concentration of verocytotoxigenic *Escherichia coli*, *Salmonella enterica* and *Listeria monocytogenes* in the beef production chain: A review. Food Microbiology. 2009;26(4):357–76. doi: 10.1016/j.fm.2008.10.012 19376457

[47] Edrington TS, Garcia Buitrago JA, Hagevoort GR, Loneragan GH, Bricta-Harhay DM, Callaway TR, et al. Effect of waste milk pasteurization on fecal shedding of Salmonella in preweaned calves. Journal of Dairy Science. 2018;101(10):9266–74. doi: 10.3168/jds.2018-14668 30077443

[48] Jacob ME, Almes KM, Shi X, Sargeant JM, Nagaraja TG. *Escherichia coli* O157:H7 Genetic Diversity in Bovine Fecal Samples. Journal of Food Protection. 2011;74(7):1186–8. doi: 10.4315/0362-028X.JFP-11-022 21740722

[49] Gragg SE, Loneragan GH, Nightingale KK, Brichta-Harhay DM, Ruiz H, Elder JR, et al. Substantial within-Animal Diversity of Salmonella Isolates from Lymph Nodes, Feces, and Hides of Cattle at Slaughter. Applied and Environmental Microbiology. 2013;79(15):4744–50. doi: 10.1128/AEM.01020-13 23793628 PMC3719521

[50] Avery LM, Killham K, Jones DL. Survival of *E*. *coli* O157:H7 in organic wastes destined for land application. Journal of Applied Microbiology. 2005;98(4):814–22. doi: 10.1111/j.1365-2672.2004.02524.x 15752326

[51] Jiang X, Morgan J, Doyle MP. Fate of *Escherichia coli* O157:H7 in Manure-Amended Soil. Applied and Environmental Microbiology. 2002;68(5):2605–9. doi: 10.1128/AEM.68.5.2605–2609.2002 11976144 PMC127522

[52] Baker CA, Lee S, De J, Jeong KC, Schneider KR. Survival of Escherichia coli O157 in autoclaved and natural sandy soil mesocosms. PLoS One. 2020;15(6):e0234562. doi: 10.1371/journal.pone.0234562 32525952 PMC7289397

[53] Xing J, Wang H, Brookes PC, Salles JF, Xu J. Soil pH and microbial diversity constrain the survival of *E*. *coli* in soil. Soil Biology and Biochemistry. 2019;128:139–49. doi: 10.1016/j.soilbio.2018.10.013

[54] Devarajan N, McGarvey JA, Scow K, Jones MS, Lee S, Samaddar S, et al. Cascading effects of composts and cover crops on soil chemistry, bacterial communities and the survival of foodborne pathogens. Journal of Applied Microbiology. 2021;131(4):1564–77. doi: 10.1111/jam.15054 33825272 PMC8519115

[55] Larney FJ, Olson AF, Miller JJ, DeMaere PR, Zvomuya F, McAllister TA. Physical and Chemical Changes during Composting of Wood Chip–Bedded and Straw-Bedded Beef Cattle Feedlot Manure. Journal of Environmental Quality. 2008;37(2):725–35. doi: 10.2134/jeq2007.0351 18396561

[56] Sidhu J, Gibbs RA, Ho GE, Unkovich I. The role of indigenous microorganisms in suppression of salmonella regrowth in composted biosolids. Water Research. 2001;35(4):913–20. doi: 10.1016/s0043-1354(00)00352-3 11235886

[57] Mallon CA, Elsas JDv, Salles JF. Microbial Invasions: The Process, Patterns, and Mechanisms. Trends in Microbiology. 2015;23(11):719–29. doi: 10.1016/j.tim.2015.07.013 26439296

[58] Tilman D, Reich PB, Isbell F. Biodiversity impacts ecosystem productivity as much as resources, disturbance, or herbivory. Proceedings of the National Academy of Sciences. 2012;109(26):10394–7. doi: 10.1073/pnas.1208240109 22689971 PMC3387045

[59] Han Z, Huang G, Liao J, Li J, Lyu G, Ma J. Disentangling survival of *Escherichia coli* O157:H7 in soils: From a subpopulation perspective. Science of The Total Environment. 2020;749:141649. doi: 10.1016/j.scitotenv.2020.141649 32829282

[60] Ma J, Ibekwe AM, Yang C-H, Crowley DE. Influence of bacterial communities based on 454-pyrosequencing on the survival of *Escherichia coli* O157:H7 in soils. FEMS Microbiology Ecology. 2013;84(3):542–54. doi: 10.1111/1574-6941.12083 23360569

[61] van Elsas JD, Chiurazzi M, Mallon CA, Elhottovā D, Krištůfek V, Salles JF. Microbial diversity determines the invasion of soil by a bacterial pathogen. Proceedings of the National Academy of Sciences. 2012;109(4):1159–64. doi: 10.1073/pnas.1109326109 22232669 PMC3268289

[62] Berlemont R, Martiny AC. Genomic Potential for Polysaccharide Deconstruction in Bacteria. Applied and Environmental Microbiology. 2015;81(4):1513–9. doi: 10.1128/AEM.03718-14 25527556 PMC4309713

[63] Flint HJ, Scott KP, Duncan SH, Louis P, Forano E. Microbial degradation of complex carbohydrates in the gut. Gut Microbes. 2012;3(4):289–306. doi: 10.4161/gmic.19897 22572875 PMC3463488

[64] Wang C, Dong D, Wang H, Müller K, Qin Y, Wang H, et al. Metagenomic analysis of microbial consortia enriched from compost: new insights into the role of Actinobacteria in lignocellulose decomposition. Biotechnology for Biofuels. 2016;9(1):22. doi: 10.1186/s13068-016-0440-2 26834834 PMC4731972

[65] Bao Y, Dolfing J, Guo Z, Chen R, Wu M, Li Z, et al. Important ecophysiological roles of non-dominant Actinobacteria in plant residue decomposition, especially in less fertile soils. Microbiome. 2021;9(1):84. doi: 10.1186/s40168-021-01032-x 33827695 PMC8028251

[66] Bennur T, Ravi Kumar A, Zinjarde SS, Javdekar V. Nocardiopsis species: a potential source of bioactive compounds. Journal of Applied Microbiology. 2016;120(1):1–16. doi: 10.1111/jam.12950 26369300

[67] Dashiff A, Junka RA, Libera M, Kadouri DE. Predation of human pathogens by the predatory bacteria Micavibrio aeruginosavorus and Bdellovibrio bacteriovorus. Journal of Applied Microbiology. 2011;110(2):431–44. doi: 10.1111/j.1365-2672.2010.04900.x 21114596

[68] Hobley L, Summers JK, Till R, Milner DS, Atterbury RJ, Stroud A, et al. Dual Predation by Bacteriophage and Bdellovibrio bacteriovorus Can Eradicate *Escherichia coli* Prey in Situations where Single Predation Cannot. Journal of Bacteriology. 2020;202(6):e00629–19. doi: 10.1128/JB.00629-19 31907203 PMC7043672

[69] Zhang W, Wang Y, Lu H, Liu Q, Wang C, Hu W, et al. Dynamics of Solitary Predation by *Myxococcus xanthus* on *Escherichia coli* Observed at the Single-Cell Level. Applied and Environmental Microbiology. 2020;86(3):e02286–19. doi: 10.1128/AEM.02286-19 31704687 PMC6974655

[70] Chen Z, Kim J, Jiang X. Survival of *Escherichia coli* O157:H7 and Salmonella enterica in animal waste-based composts as influenced by compost type, storage condition and inoculum level. Journal of Applied Microbiology. 2018;124(5):1311–23. doi: 10.1111/jam.13719 29405530

[71] Koseki S, Nakamura N, Shiina T. Comparison of Desiccation Tolerance among *Listeria monocytogenes*, *Escherichia coli* O157:H7, *Salmonella enterica*, and *Cronobacter sakazakii* in Powdered Infant Formula. Journal of Food Protection. 2015;78(1):104–10. doi: 10.4315/0362-028X.JFP-14-249 25581184

[72] Siller P, Daehre K, Thiel N, Nübel U, Roesler U. Impact of short-term storage on the quantity of extended-spectrum beta-lactamase–producing *Escherichia coli* in broiler litter under practical conditions. Poultry Science. 2020;99(4):2125–35. doi: 10.1016/j.psj.2019.11.043 32241498 PMC7587761

[73] Bartram AK, Jiang X, Lynch MDJ, Masella AP, Nicol GW, Dushoff J, et al. Exploring links between pH and bacterial community composition in soils from the Craibstone Experimental Farm. FEMS Microbiology Ecology. 2014;87(2):403–15. doi: 10.1111/1574-6941.12231 24117982

[74] Qi D, Wieneke X, Tao J, Zhou X, Desilva U. Soil pH Is the Primary Factor Correlating With Soil Microbiome in Karst Rocky Desertification Regions in the Wushan County, Chongqing, China. Frontiers in Microbiology. 2018;9. doi: 10.3389/fmicb.2018.01027 29896164 PMC5987757

[75] Rapp D, Ross C, Hea SY, Brightwell G. Importance of the Farm Environment and Wildlife for Transmission of Campylobacter jejuni in A Pasture-Based Dairy Herd. Microorganisms, 2020;8(12):1877. doi: 10.3390/microorganisms8121877 33260888 PMC7761079

