# Supplementary figures and images for: Medium-term storage of calf beddings affects bacterial community and effectiveness to inactivate zoonotic bacteria
Source: PLoS One. 2023 Dec 15;18(12):e0295843. doi: 10.1371/journal.pone.0295843 (PMC10723701; doi:10.1371/journal.pone.0295843)

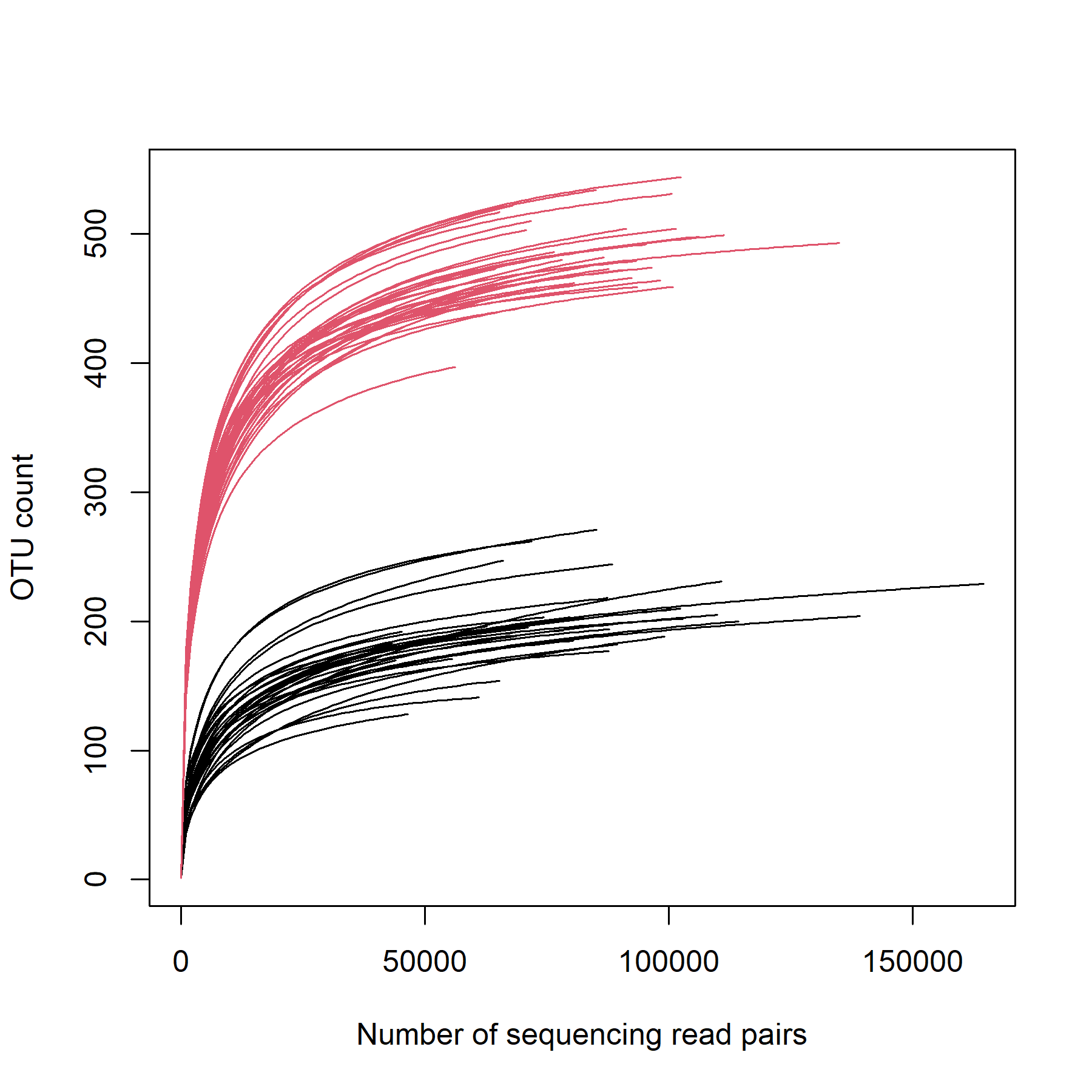

Supplement: S1 Fig — (TIFF) [file pone.0295843.s001.tiff]
